# The ICU-CARB score: a novel clinical scoring system to predict carbapenem-resistant gram-negative bacteria carriage in critically ill patients upon ICU admission

**DOI:** 10.1186/s13756-023-01326-9

**Published:** 2023-10-28

**Authors:** Yunqi Dai, Ling Zhang, Tingting Pan, Ziyun Shen, Tianjiao Meng, Jing Wu, Feifei Gu, Xiaoli Wang, Ruoming Tan, Hongping Qu

**Affiliations:** 1 Department of Critical Care Medicine, Ruijin Hospital, Shanghai Jiao Tong University School of Medicine, Shanghai, China; 2 Department of Thoracic Surgery, Shanghai Pulmonary Hospital, School of Medicine, Tongji University, Shanghai, China; 3 Department of Laboratory Medicine, Ruijin Hospital, Shanghai Jiao Tong University School of Medicine, Shanghai, China

**Keywords:** Carbapenem-resistant gram-negative bacterial, Intensive care unit, Critical patients, Scoring system, Precaution

## Abstract

**Background:**

With the widespread spread of carbapenem-resistant gram-negative bacteria (CR-GNB) in medical facilities, the carriage of CR-GNB among critically ill patients has become a significant concern in intensive care units (ICU). This study aimed to develop a scoring system to identify CR-GNB carriers upon ICU admission.

**Methods:**

Consecutive critically ill patients admitted to the ICU of Shanghai Ruijin Hospital between January 2017 and December 2020 were included. The patients were then divided into training and testing datasets at a 7:3 ratio. Parameters associated with CR-GNB carriage were identified using least absolute shrinkage and selection operator regression analysis. Each parameter was assigned a numerical score ranging from 0 to 100 using logistic regression analysis. Subsequently, a four-tier risk-level system was developed based on the cumulative scores, and assessed using the area under the receiver operating characteristic curve (AUC).

**Results:**

Of the 1736 patients included in this study, the prevalence of CR-GNB carriage was 10.60%. The clinical scoring system including seven variables (neurological disease, high-risk department history, length of stay ≥ 14 days, ICU history, invasive mechanical ventilation, gastrointestinal tube placement, and carbapenem usage) exhibited promising predictive capabilities. Patients were then stratified using the scoring system, resulting in CR-GNB carriage rates of 2.4%, 12.0%, 36.1%, and 57.9% at the respective risk levels (*P* < 0.001). Furthermore, the AUC of the developed model in the training set was calculated to be 0.82 (95% CI, 0.78–0.86), while internal validation yielded an AUC of 0.83 (95% CI, 0.77–0.89).

**Conclusions:**

The ICU-CARB Score serves as a straightforward and precise tool that enables prompt evaluation of the risk of CR-GNB carriage at the time of ICU admission, thereby facilitating the timely implementation of targeted pre-emptive isolation.

**Supplementary Information:**

The online version contains supplementary material available at 10.1186/s13756-023-01326-9.

## Introduction

Carbapenem-resistant gram-negative bacteria (CR-GNB), including carbapenem-resistant Enterobacteriaceae (CRE), carbapenem-resistant *Acinetobacter baumannii* (CRAB), and carbapenem-resistant *Pseudomonas aeruginosa* (CRPA), have emerged as significant global public health crisis [1]. Intensive care units (ICU) are a hotspot for the emergence and spread of CR-GNB owing to the complex ICU environment, critical patient conditions, frequent invasive procedures, extensive antibiotic use, and inadequate staffing [2, 3]. The limited treatment options and unfavourable outcomes associated with CR-GNB infections necessitate practical and effective preventive strategies [4, 5]. Our previous study demonstrated a substantial reduction in ICU-acquired carbapenem-resistant *Klebsiella pneumoniae* (CRKP) colonization/infection through comprehensive infection prevention and control (IPC) interventions [6–8]. According to the data from the China Antimicrobial Resistance Surveillance System (CARSS) and China Antimicrobial Surveillance Network (CHINET), the prevalence of CRKP has been showing an increasing trend from 3.0 to 30.0%, and that for CRAB from 39.0 to 79.5%; the percentage of CRPA ranged from 22.1 to 35.8% with an average rate of 22.3% in CARSS [9]. Data from Shanghai and Ruijin hospital showed a similar distribution, though a steady increase in CR-GNB carriage rates upon ICU admission has been observed since 2013 [10]. Therefore, early identification of CR-GNB carriers in the ICU can facilitate the implementation of pre-emptive isolation measures to prevent the transmission of CR-GNB among patients [11].

Currently, the primary approach to prevent and control the spread of CR-GNB infection in our country relies heavily on active surveillance culture (ASC) [12]. Guidelines recommend screening patients promptly upon admission or following exposure to CR-GNB carriers, followed by regular screening [13]. However, ASC requires traditional microbiological cultures and antibacterial susceptibility testing, leading to inevitable delays (48–72 h) between sample collection and positive culture results. To avoid cross-transmission of CR-GNB before the culture results, guidelines published in Europe and the United States recommend immediate pre-emptive isolation for known carriers of multidrug-resistant (MDR) GNB or "high-risk" patients upon admission [14, 15]. Due to their fragile pathophysiology, ICU patients often have multiple high-risk factors for MDR bacteria at the same time. Comprehensive scoring systems are used to combine various risk factors and translate them into an easily interpretable risk assessment for individuals [16]. To optimise the use of medical resources and establish scientific and standardised infection control strategies, it is crucial to develop an accurate prediction tool for CR-GNB carriers upon ICU admission [17]. This may also provide a common reference for training programmes and stratified analyses of IPC strategies using different techniques and approaches.

The aim of the present study was to identify the potential risk factors for CR-GNB carriage upon ICU admission and to develop a scoring system to facilitate the prompt identification and implementation of effective infection control measures in healthcare settings.

## Methods

### Study design and population

The characteristics and clinical information of consecutive critical patients admitted to the ICU of Shanghai Ruijin Hospital between January 2017 and December 2020 were retrospectively collected. The study was approved by the Institutional Ethics Committee of Ruijin Hospital affiliated with Shanghai Jiao Tong University School of Medicine (No. 2022-LLS-93), and informed consent was waived owing to the retrospective nature of the study.

The exclusion criteria for participants were as follows: (1) age < 18 years at the time of admission and (2) absence of ASCs within 72 h of admission. A randomization process was performed 20 times in R, each time creating a split with an approximate 7:3 ratio to generate training and validation datasets. We selected the first instance where baseline balance was achieved (All baseline variables with *P*-values > 0.05) to establish our final training and validation datasets.

ASCs, including rectal swabs, oral pharyngeal swabs, sputum samples, urine samples, and drainage cultures, were routinely conducted within 72 h of ICU admission to detect pathogen colonization/infection. CR-GNB carriage was defined as the detection of CR-GNB through ASCs and clinical cultures either before ICU admission or within 72 h after admission [18].

### Data collection and definition

All relevant data including demographic, epidemiological, clinical, biochemical, and microbiological features were retrospectively extracted from the hospital's electronic patient database. This included information on age, sex, comorbidities, prior hospitalisation, antibiotic use, surgery, and invasive therapy.

Underlying medical conditions, including neurological disease, cerebrovascular disease, coronary atherosclerotic heart disease, chronic cardiac dysfunction, chronic respiratory disease, cirrhosis, chronic kidney disease, diabetes mellitus, immunosuppression, and malignant tumours, were thoroughly documented. High-risk department history is defined as, patients with a hospitalisation history in the last 30 d at a department where carbapenem-resistant pathogens was detected in the last quarter. Neurological diseases include cerebral degeneration, Parkinson's disease, epilepsy, recurrent seizures, spinocerebellar disease, cerebellar ataxia, spinal muscular atrophy, and encephalopathy [19]. Immunosuppressed states were considered as those involving chronic corticosteroid use, immunosuppressive drug use, or human immunodeficiency virus (HIV) infection. The clinical severity of these patients upon admission was assessed using the Acute Physiology and Chronic Health Evaluation (APACHE II) and Sequential Organ Failure Assessment (SOFA) scores, calculated based on clinical and biochemical indices. Invasive therapies, vasoactive medications, and antibiotic agents administered within the first 24 h of admission, were recorded.

### Microbiological procedures

Isolates were identified using matrix-assisted laser desorption ionisation-time of flight mass spectrometer (bioMérieux, Marcy l’Etoile, France). Antimicrobial susceptibilities were determined in vitro using the VITEK 2 Compact system (bioMérieux) and disk-diffusion assays, following the guidelines set by the Clinical and Laboratory Standards Institute (CLSI) [20]. Carbapenem resistance was defined based on the CLSI interpretation, indicated as isolates resistant to imipenem, meropenem, or ertapenem. CR-GNB included CRE, CRAB, and CRPA.

### Construction of the scoring system

In the variable selection step, the Least Absolute Shrinkage and Selection Operator (LASSO) regression was applied to the training set to select the most valuable variables [21]. A nomogram for predicting CR-GNB carriage was established based on the LASSO logistic regression model. Each variable was distributed on the nomogram according to its weight, to obtain different scores. A simplified scoring system (ICU-CARB Score) was derived by assigning scores to each covariate based on the relative weight, to facilitate clinicians’ bedside interpretation.

### Validation and clinical utility

The scoring system was validated using two datasets: training and validation sets. The discrimination of the model was assessed using the receiver operating characteristic (ROC) curve and area under the ROC curve (AUC). Calibration plots were generated to assess the calibration of the model, displaying apparent (actual), bias-corrected (adjusted), and ideal (100% agreement) curves with 1000 bootstrap resamples. The clinical utility of the model was evaluated using decision curve analysis (DCA), which quantifies the net benefits at different threshold probabilities. To enhance the clinical utility of the scoring system, the risk of CR-GNB carriage was categorised into four levels (negligible, low, medium, and high risk) by quartiles of the risk score. Pearson’s contingency coefficient was used to measure the degree of association between score levels and risk of CR-GNB carriage, while the Cochran-Armitage test was used to examine trends [22].

### Statistical analysis

Statistical analyses were conducted using R statistical software, version 4.2.1 (http://cran.r-project.org). Continuous variables with a normal distribution are presented as mean and standard deviation (SD) and analysed using the Student's t-test. Continuous variables with a non-normal distribution are presented as median and interquartile range (IQR), and the Mann–Whitney U test was used for between-group analysis. Categorical variables are presented as frequencies (%) and analysed using the chi-squared test or Fisher's exact test. Two-sided *P*-values below 0.05 were considered statistically significant.

## Results

### Clinical characteristics of patients

**Fig. 1:**
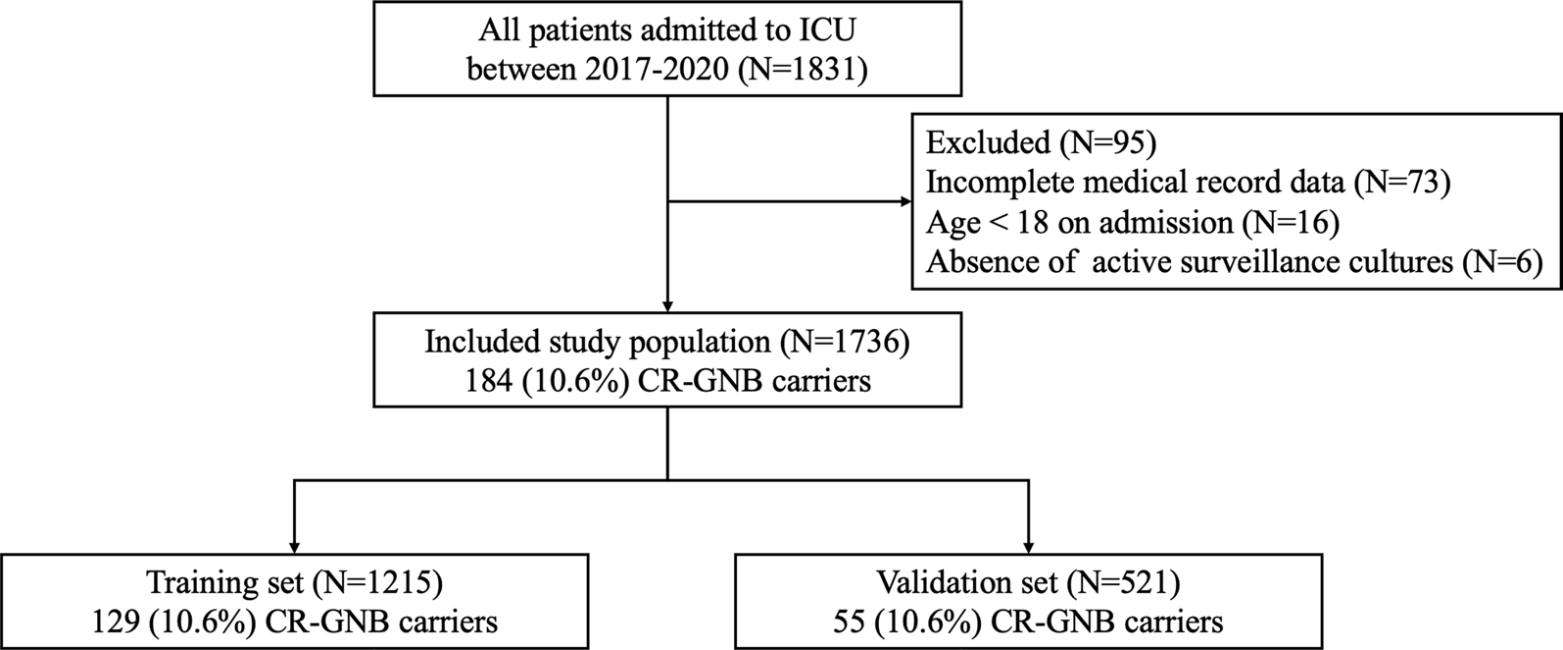
Flow chart of study participants. *CR-GNB* carbapenem-resistant gram-negative bacterial

A total of 1831 patients admitted to the ICU were initially identified for the study. After applying the exclusion criteria (Fig. 1), a final analysis was conducted on 1736 adult patients (mean age 64.1 ± 18.1 years, 724 women). Among the 1736 patients, 1143 (65.8%) and 593 (34.2%) were from the surgical and internal medicine departments, respectively. The occurrence of CR-GNB carriage upon ICU admission was 10.60% (184/1736) in the overall dataset, patient demographics and clinical characteristics stratified by CR-GNB carriage status are presented in Additional file 1: Table S1. Due to co-carriage of CR-GNB, a total of 212 strains were detected from 184 patients (Additional file 1: Table S2). It was discovered that *Acinetobacter baumannii* (38.7%) and *Klebsiella pneumoniae* (38.7%) were the most common strains, followed by *Pseudomonas aeruginosa* (13.7%), *Escherichia coli* (6.7%), *Proteus mirabilis* (0.9%), *Enterobacter cloacae* (0.5%), *Citrobacter koseri* (0.5%), and *Serratia marcescens* (0.5%). The lower respiratory tract (44.3%) is the most common site of detection, followed by rectal swab (21.7%), abdominal (13.2%), urine (7.1%), blood (4.7%), throat swab (4.2%), chest (2.8%), pus (1.4%) and catheter (0.5%).

### Risk model development

**Fig. 2:**
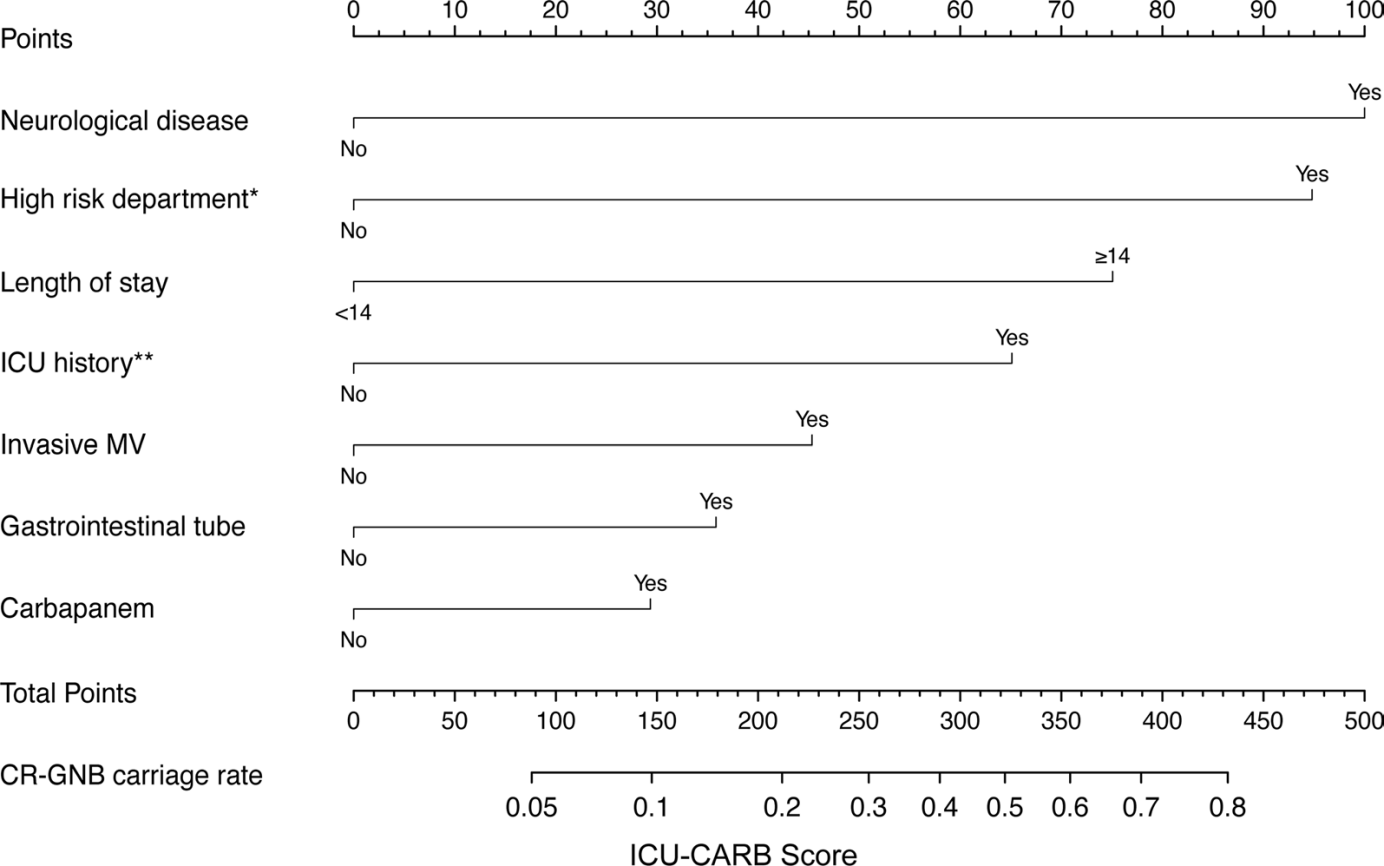
ICU-CARB Score for predicting carbapenem-resistant gram-negative bacterial carriage upon ICU admission. *A hospitalisation history in the last 30 d at departments where carbapenem-resistant pathogens were detected in the last quarter. **ICU history in the past 60 days. *ICU* intensive care unit; *MV* mechanical ventilation

The patients were randomly divided into training (n = 1215) and validation datasets (n = 521) at a ratio of 7:3. Additional file 1: Table S3 provides an overview of the basic characteristics of the two datasets. No significant differences were observed in any of the clinical characteristics between the training and validation datasets (all *P* > 0.05). Among all demographics, diseases, and treatment features, 38 variables were initially considered. Using the LASSO regression model, the variables in the training dataset were reduced to the following seven significant features: neurological disease, high-risk department history, length of stay before ICU admission, ICU history, invasive mechanical ventilation, gastrointestinal tube placement, and carbapenem administration (Additional file 1: Figure S1). These seven features exhibited non-zero coefficients in the LASSO regression model, indicating their significance in predicting CR-GNB carriage. Based on the results of the LASSO logistic regression analysis, a nomogram model (Fig. 2) was developed to predict CR-GNB carriage upon ICU admission.

### Risk score development

The scores for all variables in the ICU-CARB scoring system are presented in Table 1, and an online calculator (https://www.wjx.cn/vm/OMMYVN6.aspx) is available. The total risk score ranged from a minimum of 0 (lowest risk) to a maximum of 445 (highest risk), with the corresponding predicted probabilities of CR-GNB carriage ranging from 0.00 to 82.11%.

**Table 1:** Risk scores for all predictors

Risk factors	Score
*Neurological disease*	
No	0
Yes	100
*Transferred from high-risk department*	
No	0
Yes	95
*Length of stay before ICU*	
≥ 14 days	0
< 14 days	75
*ICU history in 60 days*	
No	0
Yes	65
*Invasive mechanical ventilation*	
No	0
Yes	45
*Gastrointestinal tube*	
No	0
Yes	36
*Carbapenem usage*	
No	0
Yes	29

*ICU* intensive care unit

### Validation of risk score

**Fig. 3:**
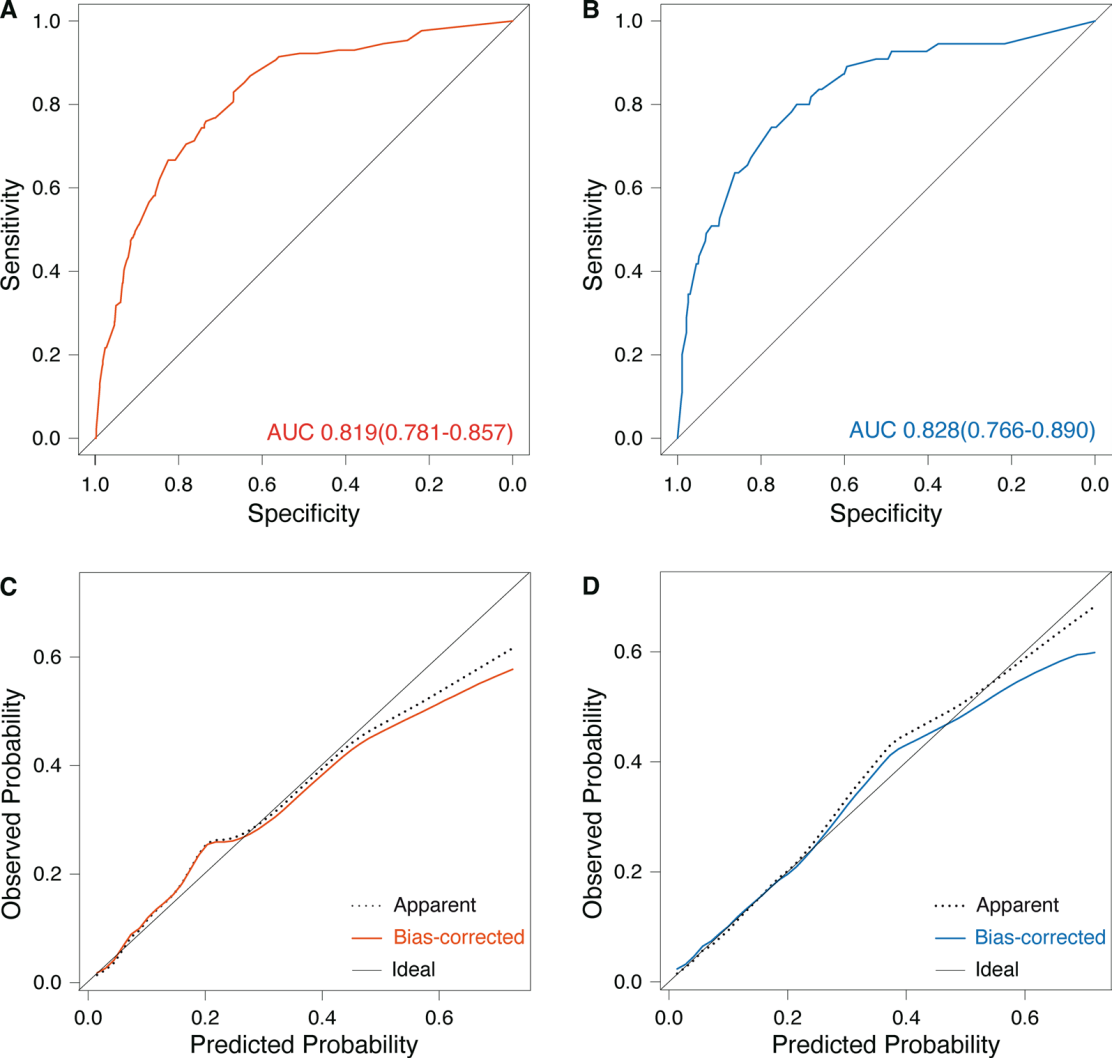
The area under the receiver operating characteristic curve (AUC) values for the prediction of CR-GNB carriage upon ICU admission in the training (**A**) and validation dataset (**B**); Calibration curve analysis in the training (**C**) and testing dataset (**D**). *CR-GNB* carbapenem-resistant gram-negative bacterial *ICU* intensive care unit

**Fig. 4:**
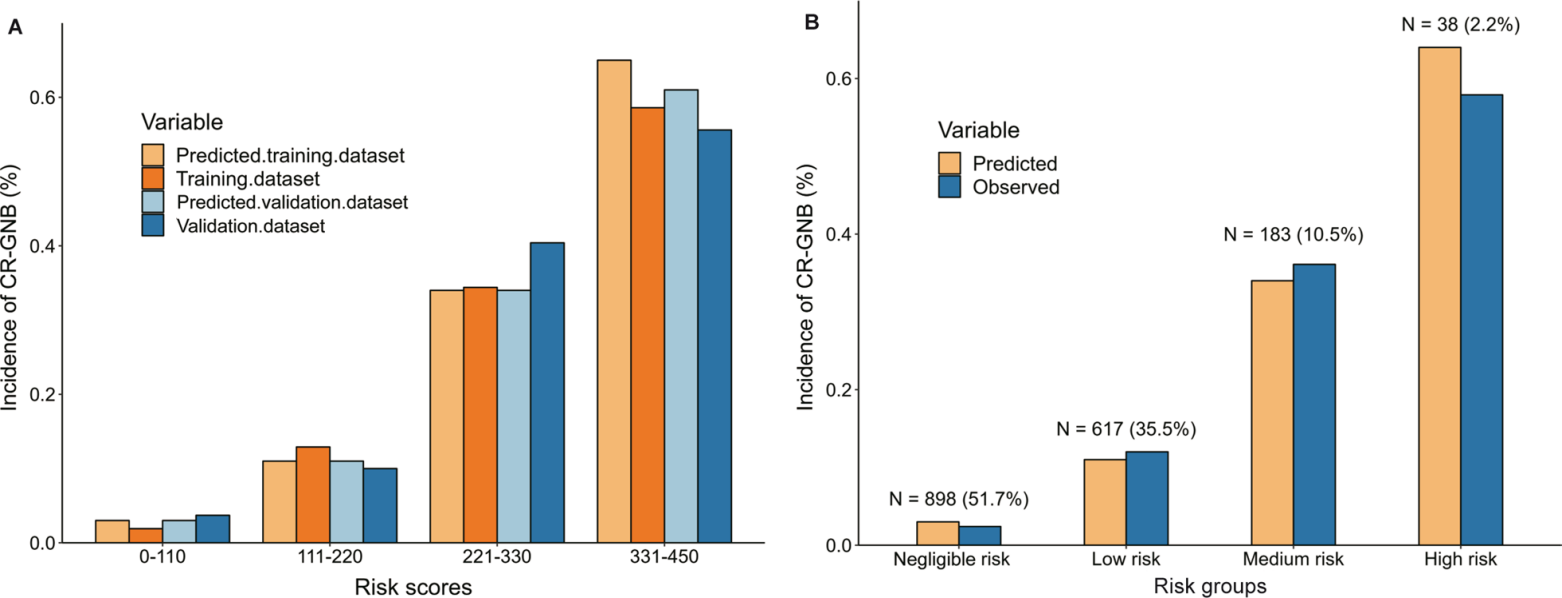
**A** Increasing risk of CR-GNB carriage upon ICU admission with increasing risk score is evident in the training and validation sets. **B** The observed incidence of CR-GNB carriage was nicely consistent with the predicted ones using ICU-CARB Score based on the data set. *CR-GNB* carbapenem-resistant gram-negative bacterial; *ICU* intensive care unit

Applying the risk score model to the training dataset gave a good discrimination, with a C-statistic of 0.819 (95% CI, 0.781–0.857) (Fig. 3A). Also, the bootstrapping internal validation yielded an average C-statistic of 0.828 (bias-corrected 95% CI, 0.766–0.890) (Fig. 3B). The ROC (Fig. 3) and prevalence of CR-GNB carriage (Fig. 4A) from both datasets were concordant.

**Fig. 5:**
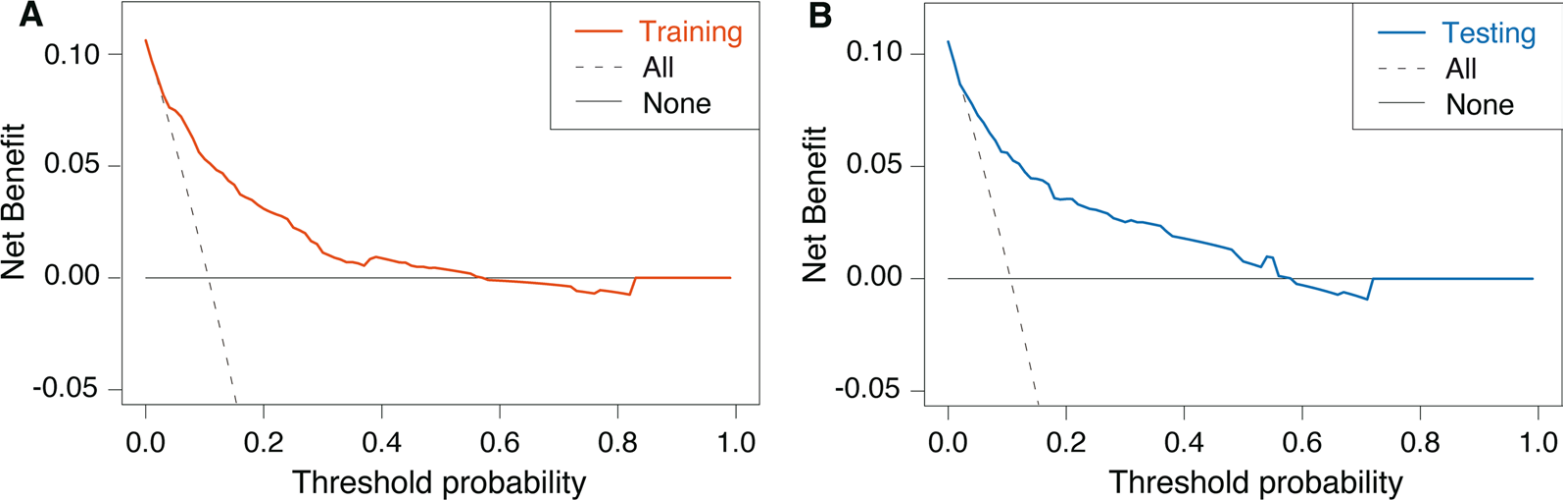
Decision curve analysis of ICU-CARB Score for predicting CR-GNB carriage upon ICU admission in the training set (**A**) and the testing set (**B**). *ICU* intensive care unit; *CR-GNB* carbapenem-resistant gram-negative bacterial

The clinical applicability of the ICU-CARB Score was assessed using DCA, as shown in Fig. 5. DCA demonstrated that within a threshold probability range of 1%–59%, utilising our risk prediction nomogram for CR-GNB carriage would yield a net benefit for patients.

### Clinical implications of the scoring system

We arbitrarily categorised the risk scores into four levels to enhance the clinical use of the scoring system. The four possible categories, negligible (score 0–110), low (111–220), medium (221–330), and high risk (331–450), are shown in Additional file 1: Table S4. The observed incidence of CR-GNB carriage (2.4%, 12.0%, 36.1%, 57.9%) was consistent with the predicted incidences (3.0%, 11.0%, 34.0%, 64.0%) using ICU-CARB Score based on the four risk allocations. As shown in Fig. 5, the risk score was highly and positively associated with the risk of CR-GNB carriage (Pearson’s contingency coefficient = 0.342; P for trend < 0.001) in both the training and validation datasets.

## Discussion

To the best of our knowledge, this is the first study to focus specifically on the predictive value of CR-GNB carriage for ICU admission. Previous studies have highlighted the significance of high SOFA scores and ICU admission as important factors associated with an increased risk of CR-GNB isolation during hospitalization [23]. Critically ill patients usually have multiple chronic comorbidities and a higher risk of organ failure, rendering them more susceptible to infection with MDR organisms. Therefore, standardised IPC strategy is needed in the ICU to reduce MDR cross-transmission [24]. Furthermore, the increasing incidence of imported CR-GNB carriers upon ICU admission poses challenges for IPC interventions, underscoring the need for proactive efforts to prevent transmission [25]. Therefore, there is an urgent need for improved methods to detect CR-GNB earlier in ICU patients.

In our study, we evaluated a total of 38 variables and identified key features that enhanced the usability of the prediction model compared with previous studies. We selected the following seven key features that were relatively easy to obtain: neurological diseases (such as cerebral degeneration, seizures, and spinocerebellar disease), transfer from departments or institutions with carbapenem-resistant pathogen detection in the last quarter, ICU history within the past 60 days, length of stay exceeding 14 days prior to ICU admission, presence of invasive mechanical ventilation, gastrointestinal tube placement, and previous carbapenem administration. The internal validation in our cohort revealed good discrimination, calibration power, and a high C-index. DCA and risk allocation demonstrated the potential for clinical application.

Several clinical risk models have been developed for predicting MDR bacterial isolation [26], but these studies often encompass a wide range of MDR-GNB without specific predictive models or with a limited focus on ICU patients [27–29]. Notably, our study specifically targeted the risk factors for CR-GNB carriage upon ICU admission. For instance, Matthaios et al. identified five independent risk factors for *K. pneumoniae* carbapenemase (KPC)-producing *K. pneumoniae* (KPC-KP) enteric colonisation upon ICU admission; however, the lack of a predictive model limits its clinical use [30]. Mario et al. developed a model for predicting KPC-KP strain isolation during hospitalisation with an AUC curve of 0.82 (95% CI, 0.80 to 0.84) [31]. Zhang et al. developed preprocedural scores for the risk of CR-GNB carriage upon admission in the gastroenterology department using seven factors. The C-statistic of this score of 0.857 slightly exceeded that of our nomogram [32]. Therefore, we aimed to elucidate the extent of this problem in departments and medical facilities prior to ICU admission. Previous research has highlighted three predictive factors as important contributors to the development of CR-GNB: history of carbapenem usage, prior ICU stay, and prolonged duration of previous hospitalisation [30]. Consistent with the findings of other studies, these factors also emerged as influential in our study. Genomic analysis revealed that the transmission rate of CRKP at the facility level is associated with its prevalence [33]. Prior studies have explored the increased risk of subsequent KPC-KP isolation in wards classified as high risk, with a 4.77 times higher risk reported [34]. Similarly, in our study, by assessing the values obtained from the nomogram, we found that previous hospitalisation history, prolonged length of stay, high-risk department history, and ICU history were the most important features for predicting CR-GNB carriage [35].

Simple and easily accessible indicators, along with an intuitive risk score, contribute to the timeliness of IPC interventions and enhance the applicability of the predictive model [36]. Although previous studies have emphasised the importance of scores such as SOFA, APACHE-II, and Pitt bacteraemia score (PBS) for prediction [37], we deliberately excluded these features because their inclusion provided negligible benefits to the predictive model. Excluding these scores did not hinder the development of an accurate model, and the inclusion of such as the APACHE II and SOFA scores, would have made our model inconvenient for clinical use [38]. Instead, all the predictive factors we considered could be readily obtained upon admission, enabling immediate identification of high-risk patients and facilitating the timely implementation of appropriate pre-emptive isolation measures.

Furthermore, we provided appropriate risk allocation based on the ICU-CARB Score, enabling ICU physicians to establish individually tailored procedures and determine the appropriate patient population for pre-emptive isolation. Unlike previously published studies, we provided an open data interface that is accessible to the public, allowing for external validation of our model. The quantitative score allows for different potential decision thresholds, and we provided four risk groups—negligible, low, medium, and high—to enable physicians to select cut-off points that align with their specific needs. This may facilitate the establishment of more tailored procedures, including individualising the patients for pre-emptive isolation.

The role of the ICU-CARB Score is to quickly identify high-risk CR-GNB carriers, and physicians can dynamically adjust IPC measures according to local conditions. In our ward, different IPC measures were implemented for patients at different risks: continuous ASC was performed for all patients during ICU stay, contact isolation was recommended for medium-risk patients, and pre-emptive isolation (single-room isolation if possible) of the high-risk group. Routine active surveillance cultures and widespread contact isolation requires more labour and expense, which are not feasible during epidemics and resource shortages. Compared with carbapenem-susceptible cases, those with CRKP, CRPA, and CRAB were associated with statistically significantly increased total hospital cost ($14,252; 4605; 7277, *P* < 0.001) and excess LOS (13.2; 5.4; 15.8 d, *P* < 0.001) [39]. In terms of the economic evaluations of IPC, implementing clinical best care practices was cost-efficient and discontinuing IPC practices was not [40, 41]. Considering possible increased costs, decisions on whether to simplify ASCs can be made based on the prediction model and local CR-GNB colonization pressure in areas with limited resources. For negligible and low-risk groups, if the colonization pressure of CR-GNB in the ward is low at that time, the frequency of ASC can be appropriately reduced. For medium and high-risk groups, performing ASC and pre-emptive isolation may reduce the total hospital cost and LOS in patients who ultimately receive additional treatment for CR-GNB infections. These suggestions remain to be verified by further research.

In addition to the internal validation, we are currently conducting a multicentre prospective study to further validate the ICU-CARB Score. To assist clinicians in utilising this model, we developed a user-friendly web-based tool that displays the risk of CR-GNB carriage and the seven important features once the variables are entered (https://www.wjx.cn/vm/OMMYVN6.aspx). These results may aid clinical decision-making and an understanding of the patients' condition, including the implementation of appropriate pre-emptive isolation strategies.

Despite these strengths, this study had several limitations. First, there may have been selection bias inherent in the retrospective design. Second, laboratory results, such as neutropenia and C-reactive protein (CRP) levels, were not included in the nomogram model owing to the study design. Third, as this was a single-centre study, there is a potential for selection bias regarding admitted patients, which might limit the generalisability of our findings. Future research should focus on the external validation of the nomogram using data from multiple centres to enhance its robustness and applicability.

## Conclusion

The newly developed risk-scoring system represents a straightforward and precise tool for early prediction of CR-GNB carriage in patients upon ICU admission. The ICU-CARB Score has potential for both clinical and research applications. The clinicians can use this tool to evaluate the risk of CR-GNB carriage prior to obtaining culture results, enabling them to promptly plan and implement the most suitable pre-emptive isolation measures.

### Supplementary Information

**Additional file 1. Table S1. **Comparison of the indicators between patients with or without CR-GNB carriage in the training set.** Table S2. **Distribution of isolated CR-GNB according to site of detection.** Table S3. **Baseline characteristics of the patients in the training set and validation set.** Table S4. **Risk levels for all patients.** Figure S1. **LASSO model coefficients of demographic and clinical feature selection using the LASSO regression model.

## Data Availability

The raw data supporting the conclusions of this article will be made available from the corresponding author, without undue reservation.

## Abbreviations

APACHE II: Acute physiology and chronic health evaluation
ASC: Active surveillance culture
AUC: Area under the receiver operating characteristic curve
CARSS: China Antimicrobial Resistance Surveillance System
CHINET: China antimicrobial surveillance network
CLSI: Clinical and Laboratory Standards Institute
CR-GNB: Carbapenem-resistant gram-negative bacteria
CRAB: Carbapenem-resistant *Acinetobacter baumannii*
CRE: Carbapenem-resistant Enterobacteriaceae
CRKP: Carbapenem-resistant *Klebsiella pneumoniae*
CRPA: Carbapenem-resistant *Pseudomonas aeruginosa*
CRP: C-reactive protein
DCA: Decision curve analysis
HIV: Human immunodeficiency virus
ICU: Intensive care units
IPC: Infection prevention and control
IQR: Interquartile range
KPC-KP: *Klebsiella pneumoniae* Carbapenemase producing *K. pneumoniae*
LASSO: Least absolute shrinkage and selection operator
MDR-GNB: Multidrug‐resistant gram‐negative bacteria
PBS: Pitt bacteraemia score
ROC: Receiver operating characteristic
SD: Standard deviation
SOFA: Sequential organ failure assessment
