# Supplementary material for: The ICU-CARB score: a novel clinical scoring system to predict carbapenem-resistant gram-negative bacteria carriage in critically ill patients upon ICU admission
Source: Antimicrob Resist Infect Control. 2023 Oct 28;12:118. doi: 10.1186/s13756-023-01326-9 (PMC10613373; doi:10.1186/s13756-023-01326-9)
Supplement: Supplementary file 1 — Additional file 1. Table S1. Comparison of the indicators between patients with or without CR-GNB carriage in the training set. Table S2. Distribution of isolated CR-GNB according to site of detection. Table S3. Baseline characteristics of the patients in the training set and validation set. Table S4. Risk levels for all patients. Figure S1. LASSO model coefficients of demographic and clinical feature selection using the LASSO regression model. [file 13756_2023_1326_MOESM1_ESM.docx]

**Supplementary table 1.** Comparison of the indicators between patients with or without CR-GNB carriage in the training set

| **Characteristic** | **CR-GNB carrier**  **(n=129)** | **No CR-GNB**  **(n=1086)** | **p** |
| --- | --- | --- | --- |
| **Demographic data** |  |  |  |
| Female (%) | 40 (31.0) | 470 (43.3) | 0.008 |
| Age (mean (SD)) | 63.29 (16.93) | 64.10 (18.24) | 0.635 |
| **Comorbid condition** |  |  |  |
| Neurological disease (%) | 10 (7.8) | 21 (1.9) | <0.001 |
| Cerebrovascular disease (%) | 15 (11.6) | 111 (10.2) | 0.620 |
| Coronary atherosclerotic heart disease (%) | 18 (14.0) | 145 (13.4) | 0.850 |
| Chronic heart failure (%) | 159 (13.1) | 79 (15.2) | 0.249 |
| Chronic respiratory disease (%) | 5 (3.9) | 59 (5.4) | 0.454 |
| Chronic kidney disease (%) | 10 (7.8) | 69 (6.4) | 0.543 |
| Diabetes mellitus (%) | 18 (14.0) | 208 (19.2) | 0.151 |
| Cirrhose (%) | 1 (0.8) | 32 (2.9) | 0.151 |
| Immunosuppressed state (%) | 4 (3.1) | 16 (1.5) | 0.170 |
| Malignant tumor (%) | 60 (46.5) | 587 (54.1) | 0.105 |
| **Previous hospitalization data** |  |  |  |
| LOS before ICU admission ≥14 (%) | 70 (54.3) | 241 (22.2) | <0.001 |
| Previous ICU admission (%) ^a^ | 45 (34.9) | 75 (6.9) | <0.001 |
| Previous surgery (%) ^b^ | 85 (65.9) | 799 (73.6) | 0.064 |
| Previous emergency surgery (%) ^b^ | 37 (28.7) | 160 (14.7) | <0.001 |
| Previous abdominal surgery (%) ^b^ | 61 (47.3) | 575 (52.9) | 0.224 |
| High-risk departments history (%) ^b^ | 97 (75.2) | 384 (35.4) | <0.001 |
| **Admission data** |  |  |  |
| APACHE.II (median [IQR]) | 17 [14, 23] | 14 [10, 20] | <0.001 |
| SOFA (median [IQR]) | 6 [4, 9] | 4 [2, 8] | <0.001 |
| Invasive mechanical ventilation (%) | 83 (64.3) | 431 (39.7) | <0.001 |
| Renal replacement therapy (%) | 15 (11.6) | 43 (4.0) | <0.001 |
| Central venous catheter (%) | 99 (76.7) | 746 (68.7) | 0.060 |
| Gastrointestinal tube (%) | 88 (68.2) | 430 (39.6) | <0.001 |
| Drainage (%) | 77 (59.7) | 665 (61.2) | 0.734 |
| Abdominal drainage (%) | 62 (48.1) | 521 (48.0) | 0.985 |
| Thoracic drainage (%) | 20 (15.5) | 69 (6.4) | <0.001 |
| Norepinephrine (%) | 35 (27.1) | 217 (20.0) | 0.058 |
| Epinephrine (%) | 5 (3.9) | 38 (3.5) | 0.827 |
| Dopamine (%) | 5 (3.9) | 32 (2.9) | 0.561 |
| **Antibiotic administration ^c^** |  |  |  |
| Antibiotics (%) | 108 (83.7) | 769 (70.8) | 0.002 |
| Carbapenem (%) | 77 (59.7) | 365 (33.6) | <0.001 |
| β-lactams/β-lactamase inhibitors (%) | 19 (14.7) | 89 (8.2) | 0.014 |
| 3^rd^ and 4^th^ generation cephalosporins (%) | 0 (0.0) | 2 (0.2) | 0.626 |
| Quinolone (%) | 5 (3.9) | 58 (5.3) | 0.478 |
| Oxazolidinones (%) | 20 (15.5) | 101 (9.3) | 0.026 |
| Antifungal agent (%) | 9 (7.0) | 41 (3.8) | 0.084 |

CR-GNB, Carbapenem-resistant Gram-negative bacteria; LOS, length of stay; APACHE, acute physiology and chronic health evaluation; SOFA, sequential organ failure assessment.

^a^ Administration for more than 3 days within last 2 months prior to ICU admission

^b^ Within last month prior to ICU admission.

^c^ Only intravenous administration of antibiotics in patients is included.

**Supplementary Table 2.** Distribution of isolated CR-GNB according to site of detection

|  | **No. (%) of isolates** | **No. of isolates detected from the site** | | | | | | | | |
| --- | --- | --- | --- | --- | --- | --- | --- | --- | --- | --- |
| **Organism** |  | **lower respiratory tract** | **rectal swab** | **abdominal** | **urine** | **blood** | **throat swab** | **pleural cavity** | **abscess** | **catheter** |
| CR-GNB carriage | 212 | 94 | 46 | 28 | 15 | 10 | 9 | 6 | 2 | 1 |
| *A. baumannii* | 82 (38.7) | 55 | 6 | 10 | 4 | 1 | 4 | 2 | 0- | 0 |
| *K. pneumoniae* | 82 (38.7) | 19 | 32 | 12 | 4 | 5 | 5 | 1 | 2 | 1 |
| *P. aeruginosa* | 29 (13.7) | 18 | 2 | 1 | 4 | 1 | 0 | 3 | 0 | 0 |
| *E. coli* | 14 (6.7) | 0 | 5 | 4 | 3 | 2 | 0 | 0 | 0 | 0 |
| Other CR-GNB ^a^ | 5 (2.4) | 2 | 1 | 1 | 0 | 1 | 0 | 0 | 0 | 0 |

CR-GNB, Carbapenem-resistant Gram-negative bacteria.

^a^ Other CR-GNB isolates included *Proteus mirabili* (n = 2), *Enterobacter cloacae* (n = 1), *Serratia marcescens* (n = 1), and *Citrobacter koseri* (n = 1).

**Supplementary table 3.** Baseline characteristics of the patients in the training set and validation set

| **Characteristic** | **Training set**  **(n=1215)** | **Validation set**  **(n=521)** | **p** |
| --- | --- | --- | --- |
| CR-GNB carrier (%) | 129 (10.6) | 55 (10.6) | 0.970 |
| **Demographic data** |  |  |  |
| Female (%) | 705 (58.0) | 318 (61.0) | 0.242 |
| Age (mean (SD)) | 64.01 (18.10) | 64.31 (18.19) | 0.749 |
| **Comorbid condition** |  |  |  |
| Neurological disease (%) | 31 (2.6) | 9 (1.7) | 0.294 |
| Cerebrovascular disease (%) | 126 (10.4) | 62 (11.9) | 0.347 |
| Coronary atherosclerotic heart disease (%) | 163 (13.4) | 78 (15.0) | 0.390 |
| Chronic heart failure (%) | 159 (13.1) | 79 (15.2) | 0.249 |
| Chronic respiratory disease (%) | 64 (5.3) | 28 (5.4) | 0.927 |
| Chronic kidney disease (%) | 79 (6.5) | 31 (6.0) | 0.665 |
| Diabetes mellitus (%) | 226 (18.6) | 106 (20.3) | 0.397 |
| Cirrhose (%) | 33 (2.7) | 17 (3.3) | 0.532 |
| Immunosuppressed state (%) | 20 (1.6) | 11 (2.1) | 0.502 |
| Malignant tumor (%) | 647 (53.3) | 289 (55.5) | 0.395 |
| **Previous hospitalization data** |  |  |  |
| LOS before ICU admission ≥14 (%) | 311 (25.6) | 122 (23.4) | 0.336 |
| Previous ICU admission (%) ^a^ | 120 (9.9) | 42 (8.1) | 0.233 |
| Previous surgery (%) ^b^ | 884 (72.8) | 377 (72.4) | 0.865 |
| Previous emergency surgery (%) ^b^ | 197 (16.2) | 91 (17.5) | 0.520 |
| Previous abdominal surgery (%) ^b^ | 636 (52.3) | 280 (53.7) | 0.593 |
| High-risk departments history (%) ^b^ | 481 (39.6) | 200 (38.4) | 0.639 |
| **Admission data** |  |  |  |
| APACHE.II (median [IQR]) | 15 [11, 21] | 15 [11, 21] | 0.799 |
| SOFA (median [IQR]) | 5.00 [2, 8] | 5 [3, 8] | 0.185 |
| Invasive mechanical ventilation (%) | 514 (42.3) | 238 (45.7) | 0.193 |
| Renal replacement therapy (%) | 58 (4.8) | 21 (4.0) | 0.496 |
| Central venous catheter (%) | 845 (69.5) | 359 (68.9) | 0.791 |
| Gastrointestinal tube (%) | 518 (42.6) | 239 (45.9) | 0.212 |
| Drainage (%) | 742 (61.1) | 323 (62.0) | 0.716 |
| Abdominal drainage (%) | 583 (48.0) | 262 (50.3) | 0.379 |
| Thoracic drainage (%) | 89 (7.3) | 43 (8.3) | 0.504 |
| Norepinephrine (%) | 252 (20.7) | 114 (21.9) | 0.593 |
| Epinephrine (%) | 43 (3.5) | 21 (4.0) | 0.618 |
| Dopamine (%) | 37 (3.0) | 13 (2.5) | 0.530 |
| **Antibiotic administration ^c^** |  |  |  |
| Antibiotics (%) | 877 (72.2) | 383 (73.5) | 0.569 |
| Carbapenem (%) | 442 (36.4) | 194 (37.2) | 0.734 |
| β-lactams/β-lactamase inhibitors (%) | 108 (8.9) | 37 (7.1) | 0.217 |
| 3^rd^ and 4^th^ generation cephalosporins (%) | 2 (0.2) | 2 (0.4) | 0.383 |
| Quinolone (%) | 63 (5.2) | 31 (6.0) | 0.519 |
| Oxazolidinones (%) | 121 (10.0) | 59 (11.3) | 0.392 |
| Antifungal agent (%) | 50 (4.1) | 17 (3.3) | 0.398 |

CR-GNB, Carbapenem-resistant Gram-negative bacteria; LOS, length of stay; APACHE, acute physiology and chronic health evaluation; SOFA, sequential organ failure assessment.

^a^ Administration for more than 3 days within last 2 months prior to ICU admission

^b^ Within last month prior to ICU admission.

^c^ Only intravenous administration of antibiotics in patients is included.

**Supplementary table 4.** Risk levels for all patients

| **Risk level** | **Score** | **Patients** | **Predicted risk (%)** | **Observed risk (%)** |
| --- | --- | --- | --- | --- |
| Negligible risk | 0-110 | 898 (51.7) | 3 | 2.4 |
| Low risk | 111-220 | 617 (35.5) | 11 | 12.0 |
| Medium risk | 221-330 | 183 (10.5) | 34 | 36.1 |
| High risk | 331-450 | 38 (2.2) | 64 | 57.9 |

**Supplementary figure 1.** LASSO model coefficients of demographic and clinical feature selection using the LASSO regression model. LASSO: Least absolute shrinkage and selection operator
